# Development of Non-HLA Antibodies and Their Association With Antibody-Mediated Rejection in Pediatric Kidney Transplant Recipients

**DOI:** 10.3389/ti.2025.14463

**Published:** 2025-08-07

**Authors:** Franziska Schmidt, Murielle Verboom, Michael Hallensleben, Alexander Braumann, Jens Drube, Lena Brunkhorst, Dieter Haffner, Anette Melk, Nele Kanzelmeyer

**Affiliations:** ^1^ Department of Pediatric Kidney, Liver, Metabolic and Neurological Diseases, Hannover Medical School, Hannover, Germany; ^2^ Institute of Transfusion Medicine and Transplant Engineering, Hannover Medical School, Hannover, Germany; ^3^ Institute for Mathematical Stochastics, Technische Universität Braunschweig, Braunschweig, Germany

**Keywords:** non-HLA antibodies, antibody-mediated rejection, pediatric kidney transplantation, graft loss, pediatric kidney failure

## Abstract

Antibody-mediated rejection (ABMR) is the leading cause of long-term graft loss in pediatric kidney transplantation (KTx). While donor-specific HLA antibodies are established contributors, emerging evidence suggests a role for non-HLA antibodies in ABMR pathogenesis. In this descriptive study, we analyzed 60 non-HLA antibodies in 77 pediatric KTx recipients using serum samples collected pre-transplant, post-transplant, and at ABMR diagnosis. During a median follow-up of 4.83 years, 29.8% developed ABMR, with a median onset of 3.67 years. Non-HLA antibody presence prior to KTx was not influenced by pre-transplant dialysis; over half of the patients already had >15 positive non-HLA antibodies. The cumulative antibody profile remained stable 1–2 years post-KTx, with no association between late ABMR and antibody strength or breadth. However, ACTIN (higher risk) and CGB5 (lower risk) at 1–2 years post-KTx, as well as SNRPB2 pre-transplant, were significantly associated with ABMR (p < 0.05). IL-21 levels increased in controls over time (p < 0.05), although driven by five patients with notably high levels. Our findings support a potential involvement of non-HLA antibodies in pediatric ABMR. Nevertheless, larger studies are needed to validate the predictive value of individual non-HLA antibodies for clinical application.

## Graphical Abstract

**Figure:**
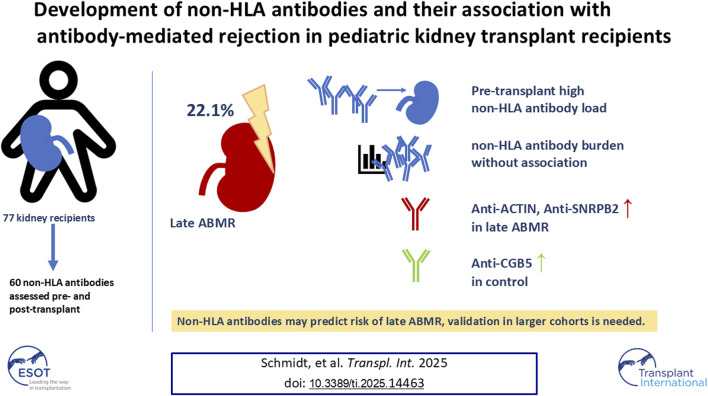

## Introduction

Kidney transplantation (KTx) is regarded as the treatment of choice for children and adolescents with kidney failure [1–4]. Antibody-mediated rejection (ABMR) remains a leading cause of graft dysfunction and loss of allograft in both pediatric and adult kidney transplant recipients [5–8]. In a study of 337 pediatric KTx patients, 12.5% developed ABMR, of which 45% of the children experienced ABMR within 5 years after KTx. Twenty-five percent of the affected children experienced graft loss within 1 year following the diagnosis of acute ABMR, while approximately 50% of patients lost their graft within 1 year after chronic ABMR diagnosis [5].

While some risk factors for adults are also applicable to children, there are age-specific aspects, including immunological naivety in young children and difficulties with adherence to immunosuppressive therapy in adolescents [9–11]. Donor-specific antibodies (DSAs) directed against human leukocyte antigens (HLAs) have been identified as pivotal targets in the humoral immune response to renal allografts, contributing to antibody-mediated rejection [5, 12]. Nevertheless, the precise underlying causal mechanisms of these associations remain unclear [13–15]. Numerous studies have identified non-HLA antibodies as potential mediators of transplant rejection [16, 17].

Non-HLA antibodies play a complementary role in allo- and autoimmunity [18–20]. Their development is initiated by endothelial injury and subsequent exposure to neoantigens or polymorphic antigens differing between donor and recipient. Moreover, cells of the basal membrane and extracellular matrix exposed by vascular injuries can activate immunological processes. Consequently, a considerable number of non-HLA antibodies associated with transplant rejection are directed against antigens on endothelial cells, as well as transmembrane and extracellular proteins [18, 19]. In addition to vascular injuries, mismatches of non-HLA antigens between donor and recipient can increase the development of non-HLA alloantibodies. Among non-HLA antibodies, antibodies against MHC class I polypeptide-related sequence A (MICA) and autoantigens such as angiotensin II receptor type 1 (AT1R), endothelin-1 receptor type A (ETAR), and vimentin have recently been in research focus [21–28]. Non-HLA antibodies are implicated in autoimmune processes, with anti-AT1R and ETAR antibodies identified in several vasculopathic conditions, including peripheral arterial occlusive disease and essential hypertension [29–31]. In fact, anti-AT1R antibodies are detectable in 20%–40% of patients with kidney failure, yet only in 7%–15% of healthy individuals [23, 24].

It has been observed that 72% of pediatric patients with DSAs against the HLA surface antigens of the donor kidney additionally exhibited non-HLA antibodies [32]. Regardless of HLA-DSAs, the presence of AT1R antibody, ETAR antibody, and MICA antibody in pediatric kidney transplant recipients is associated with an elevated risk of acute ABMR and deterioration of graft function [32]. The incidence of acute ABMR was found to be approximately 3% higher in cases where non-HLA antibodies, particularly those directed against MICA, were identified prior to KTx [33]. Nevertheless, a considerably broader spectrum of non-HLA antibodies targeting endothelial and epithelial cells, in addition to various proteins, has been associated with unfavorable outcomes in kidney transplantation [34–36]. The role of non-HLA antibodies in the context of the developing immune system in pediatric patients remains poorly characterized [37, 38]. The existing literature indicates that non-HLA antibodies influence the trajectory of allograft function in pediatric KTx patients. Comprehensive studies, particularly those involving very young transplant recipients, are still required to elucidate the interplay between non-HLA antibodies and the onset of ABMR.

To fill these gaps, we investigated the development of non-HLA antibodies and their association with ABMR in a cohort of 77 pediatric KTx patients.

## Materials and Methods

### Study Design and Study Population

We conducted a retrospective descriptive cohort study including pediatric patients with the following inclusion criteria: a) age <18 years at time of KTx, b) KTx performed at Hannover Medical School between May 2014 and June 2021, c) availability of pre- and post-transplant biobanked serum samples for HLA-DSA analysis. Exclusion criteria were defined as: a) combined organ transplantation, b) graft loss due to recurrence of the underlying disease, or c) loss to follow-up. Donor-recipient matching prior to KTx was based on blood group and HLA typing. The majority of patients received tacrolimus, mycophenolic acid or everolimus, and corticosteroids as baseline immunosuppression. Basiliximab induction was administered in patients treated with tacrolimus, prednisolone, and everolimus (initiated 4 weeks post-KTx). HLA antibodies were assessed annually and upon biopsy indication. ABMR was diagnosed via kidney allograft biopsy, which was performed when serum creatinine increased rapidly or progressively by >20% above baseline without an alternative explanation or as part of a surveillance protocol six or more months post-transplantation. Biopsies were screened ABMR according to the most recent available Banff consensus [39]. Rejection within the first-year post-transplant was defined as early ABMR; later on as late ABMR. Patients harboring histological abnormalities apart from ABMR, such as borderline, T-cell-mediated rejections, or inconclusive results, are included in the control group. Patients diagnosed with biopsy-proven ABMR received anti-humoral therapy, including plasmapheresis, intravenous immunoglobulin G, and rituximab. Non-HLA antibodies were analyzed retrospectively from biobanked serum samples obtained at several stages: pre-transplant (taken for the last evaluation of HLA alloantibodies before KTx), and post-transplant (twice within the first 2 years after KTx and whenever a kidney biopsy was performed).

Parents’ and, if appropriate, patients’ consent and the ethics committee approval of the Hannover Medical School were obtained and all investigations were performed in accordance with the Declaration of Helsinki and the Good Clinical Practice guidelines.

### Detection of Non-HLA Antibodies

The pre- and post-transplant serum samples were retrospectively analyzed for 60 non-HLA antibodies using the LIFECODES non-HLA autoantibody assay (Werfen), performed according to manufacturer’s instructions. Raw data were collected via Luminex 200 with hlaSYSTEM software (AVALAS). After background fluorescence subtraction, ratios of the measured Mean Fluorescence Intensity (MFI) to vendor-defined cut-offs were calculated for each non-HLA target; antibodies were defined as positive if ratios exceeded 1.0.

### Statistical Analysis

Descriptive statistics for categorical variables were reported as frequencies and percentages; continuous variables were summarized using means with standard deviations or medians and interquartile ranges, as appropriate. Baseline characteristics included sex, age, donor type, pre-transplant dialysis or preemptive transplantation status, *de novo* versus repeat transplantation, and the presence of DSAs pre-transplant and at routine follow-up (1–2 years post-transplant). For each patient, the broadness, number of positive non-HLA antibody targets with ratio >1, and the strength of non-HLA sensitization, sum of positive antibody ratios with a value >1 per patient, were calculated. Group differences were analyzed using Student’s t-test for normally distributed data and Wilcoxon signed-rank test otherwise. Differences between more than two groups were assessed by ANOVA. Fisher’s exact test was used for associations between categorical variables. Pearson correlation analyses assessed the relationship between identical non-HLA antibodies pre-transplant and 1–2 years post-transplant, and between different non-HLA antibodies 1–2 years post-transplant (weak or no correlation: r <0.4; moderate: r = 0.4–0.8; strong: r >0.8). Temporal antibody dynamics were evaluated by mean MFI ratios per group at four time points: pre-transplant, up to 1 year post-transplant, 1–2 years post-transplant, and at rejection. Patients were classified as controls or ABMR cases, with or without HLA-DSA at rejection. For each group, the five antibodies with the greatest net increase were identified by averaging absolute changes in MFI ratios between pre-transplant and either 1–2 years post-transplant or time of rejection, irrespective of direction. Multiple logistic regression analyzed associations between late ABMR occurrence, baseline characteristics, and cumulative antibody broadness and strength. Individual non-HLA antibody associations were assessed via L1-penalized logistic regression [40]. The hyperparameter of the L1-penalization was determined by cross-validation and AIC was used for the stepwise selection algorithm. Statistical significance was defined as p < 0.05. Throughout all analyses, the control group was defined as patients who never fulfilled diagnostic criteria for ABMR during follow-up.

## Results

### Demographic Characteristics of the Study Population

A total of 77 patients were eligible for further analysis in this study. The mean age at the time of transplantation was 10.06 years, with a male predominance (61.04%), Table 1. Most underlying conditions were renal, with congenital and genetic kidney diseases accounting for 75%, including CAKUT (n = 32) as the most frequent subtype, (Table 2). Degenerative nephritic or nephrotic disorders were observed in 13%. Living donor transplants were performed in 21%, and 19% were re-transplantations. Prior to KTx, 56% of patients underwent dialysis. Median follow-up was 4.83 years (IQR 3.08–6.96).

**TABLE 1:** Baseline characteristics for the different patient groups (total, control and ABMR, further categorized as early onset, late onset an early and late onset.

Baseline characteristics								
			Total	Control	Early ABMR	Late ABMR	Early and late ABMR*	p
Variables			n = 77	n = 54	n = 5	n = 17	n = 1	
Sex	f	n/total	30/77	20/54	1/5	8/17	1/1	0.53
		(%)	(38.98)	(37.04)	(20)	(47.06)	(100)	
	m	n/total	47/77	34/54	4/5	9/17	0/1	
		(%)	(61.04)	(62.96)	(80)	(52.94)	(0)	
Age at time of KTx		mean	10.06	9.5	15	10	6	0.06
		+/− SD	+/−5.37	+/−5.6	+/− 0.8	+/− 4.39	+/− 0	
Dialysis prior to KTx	yes	n/total	43/77	33/54	2/5	9/17	0/1	0.60
		(%)	(55.84)	(61.11)	(40)	(52.94)	(100)	
	no	n/total	34/77	21/54	3/5	8/17	1/1	
		(%)	(44.16)	(38.89)	(60)	(47.06)	(0)	
Donor type	LD	n/total	16/77	13/54	2/5	0/17	1/1	0.05
		(%)	(20.78)	(24.07)	(40)	(0)	(0)	
	DD	n/total	61/77	41/54	3/5	17/17	0/1	
		(%)	(79.22)	(75.93)	(60)	(100)	(100)	
Repeated KTx	yes	n/total	62/77	44/54	5/5	13/17	0/1	0.50
		(%)	(80.52)	(81.15)	(100)	(76.47)	(100)	
	no	n/total	15/77	10/54	0/5	4/17	1/1	
		(%)	(19.48)	(18.52)	(0)	(23.53)	(0)	
DSA pre KTx	yes	n/total		2/54	0/5	3/17	0/1	0.11
		(%)		(3.7)	(0)	(23.53)	(100)	
	no	n/total		52/54	4/5	14/17	1/1	
		(%)		(96.3)	(100)	(76.47)	(0)	
DSA after 1–2 years post KTx	yes	n/total		0/54	1/5	8/17	0/1	<0.001
		(%)		(0)	(20)	(47.06)	(100)	
	no	n/total		53/54	3/5	9/17	1/1	
		(%)		(98.15)	(60)	(52.94)	(0)	
	n/a^a^	n/total		1/54	1/5	0/17	0/17	
		(%)		(1.9)	(20)	(0)	(0)	

Abbreviations: p, p-value; f, female; m, male; KTx, kidney transplantation; LD, living donor; DD, deceased donor; n/a, not available.

^a^ Excluded from ANOVA, analysis due to low number of cases.

**TABLE 2:** Underlying causes of end-stage kidney disease in the study cohort, (N = 75).

Etiology	Diagnosis	Number of patients
Congenital and Genetic Kidney Diseases (75%)	CAKUT (Congenital anomalies of the kidney and urinary tract)	32
	Nephronophthisis	6
	Cystinosis	4
	Denys-Drash syndrome	4
	ARPKD (Autosomal recessive polycystic kidney disease)	3
	Joubert syndrome	3
	Primary hyperoxaluria type 1	3
	aHUS (Atypical hemolytic uremic syndrome)	3
	Congenital nephrotic syndrome	2
	Prune-Belly syndrome with bilateral renal dysplasia	1
	Mayer-Rokitansky-Küster-Hauser syndrome	1
	Renal coloboma syndrome	1
	Renal hypoplasia associated with branchio-oto-renal syndrome	1
	Spina bifida with neurogenic bladder	1
	Nephrocalcinosis	1
Degenerative Nephritic and Nephrotic Conditions (13%)	FSGS (Focal segmental glomerulosclerosis)	3
	Rapidly progressive glomerulonephritis	2
	Nephrotic syndrome (secondary ESRD)	2
	Tubulointerstitial nephritis	2
	Anti-GBM glomerulonephritis	1
Secondary Non-Renal Cause (1%)	Post cardiopulmonary bypass surgery (heart-lung machine operation)	1

During the follow-up period, 23 patients (29.87%) were diagnosed with ABMR: five early (6.5%), 17 late (22.1%) one with early and late ABMR (1.3%). Late ABMR occurred after a median of 6.1 years, IQR (3.68–6.41). Within the late ABMR group, 52.9% of patients exhibited the presence of HLA-DSA antibodies at the onset of rejection. Pre-transplant DSA were more frequent in late ABMR (23.5%) than controls (3.7%), and 1–2 years post-transplant, DSA were detected in 47.1% of late ABMR patients versus none in controls (p < 0.05). Donor type also differed significantly between groups. No other significant differences were observed for these characteristics between those who developed early, late or both forms of ABMR and the control group, (Table 1).

In the control group, indication biopsies were performed in 41 of 54 patients (76%) within 2 years post-transplantation. In cases with multiple histopathological findings, the predominant lesion was used for classification, (Supplementary Table S1). In 48.8% of the cases and thus most prominent were findings consistent with chronic allograft dysfunction, including tubular atrophy, interstitial fibrosis, and calcineurin inhibitor (CNI) toxicity. T cell–mediated rejection and borderline changes were observed in 31.7% of cases (n = 8 and n = 5, respectively).

### Strength and Broadness of Non-HLA Antibodies

**FIGURE 1:**
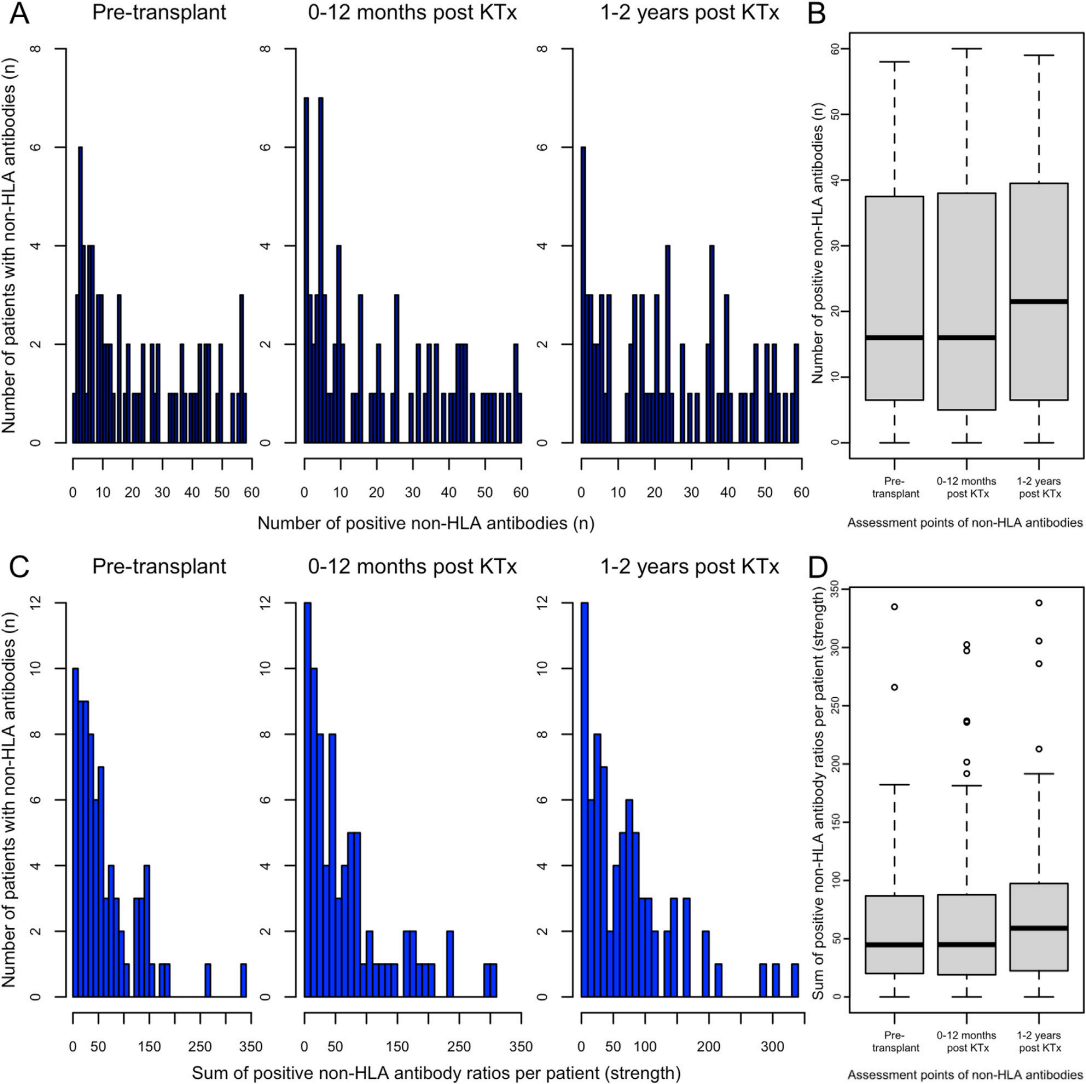
Distribution of patients by the number of positive pre-transplant non-HLA antibodies. **(A)** The broadness of the non-HLA antibody profile, defined as the count of positive non-HLA antibodies per patient, is shown for the following periods: pre-transplant, 1–12 months, and 1–2 years post-transplant (from left to right). **(B)** ANOVA on differences between the broadness of the non-HLA antibodies pretransplant, 1–12 months and 1–2 years post KTx (from left to right). **(C)** The strength, defined as the sum of all non-HLA antibody ratios >1 per patient (total positive ratio), is shown for the following periods: pre-transplant, 1–12 months, and 1–2 years post-transplant (from left to right), bars represent categories constructed in 10-unit increments. **(D)** ANOVA on differences between the cumulative strength of the non-HLA antibodies pretransplant, 1–12 months and 1–2 years post KTx (from left to right).

The distribution of positive non-HLA antibodies per patient, reflecting the broadness of non-HLA immunity, is shown in Figure 1A; cumulative proportions of all positive non-HLA antibodies per patient, indicating the total strength of the non-HLA antibody response, are shown in Figure 1B. Two patients were excluded from analysis due to missing values. Data are presented for three time periods: pre-transplant, up to 1 year post-transplant, and 1–2 years post-transplant. At pre-transplant stage, 53% of patients (40/75) demonstrated more than 15 positive non-HLA antibodies. Although a tendency toward an expanded spectrum of positive non-HLA antibodies was observed between 1 and 2 years post-transplant, this shift did not reach statistical significance. Furthermore, no significant changes were observed in the cumulative strength of positive non-HLA antibodies throughout the post-transplant period.

**FIGURE 2:**
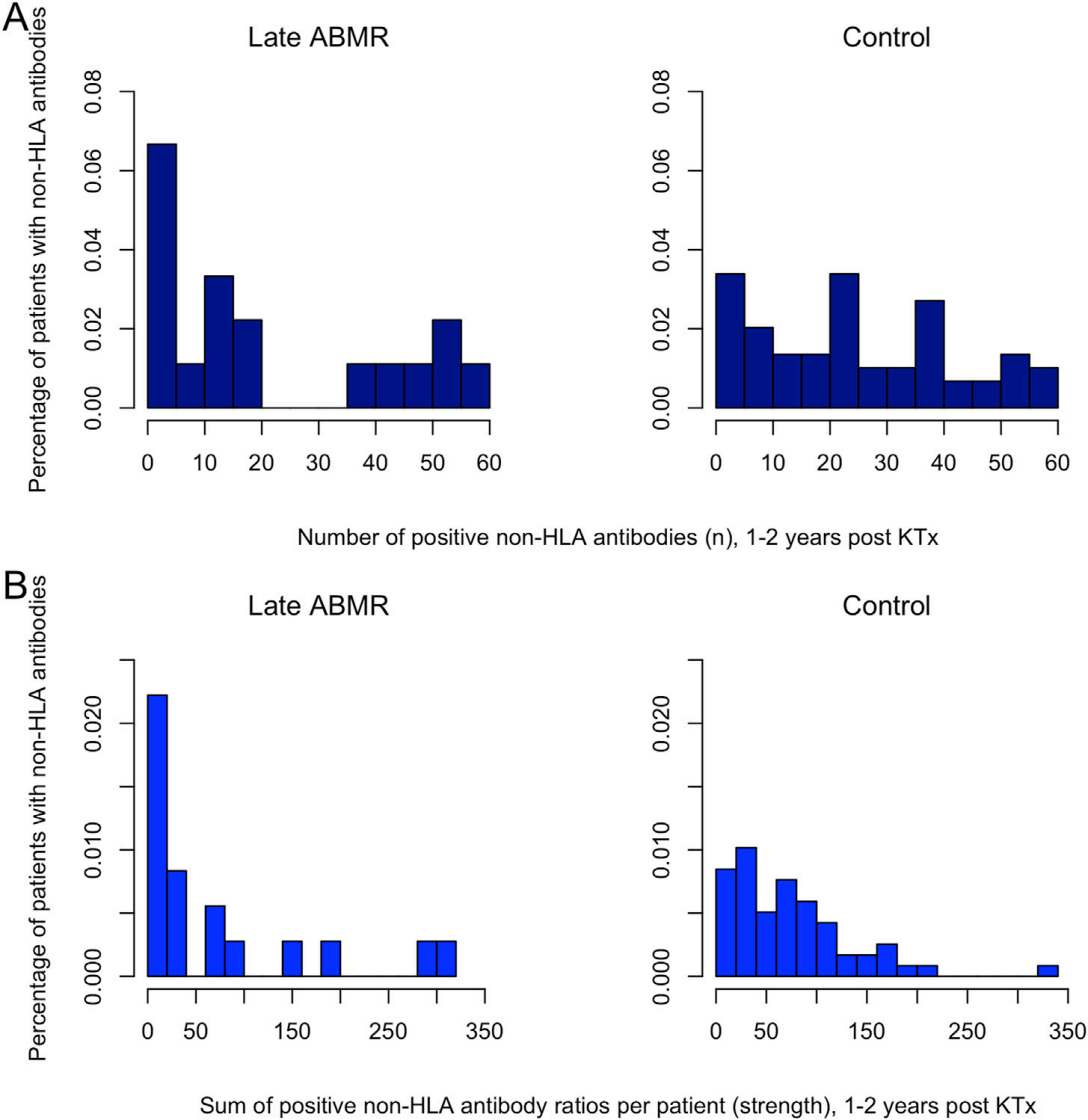
Distribution of the relative frequencies of broadness and strength in the non-HLA antibody profile. **(A)** Distribution of the relative frequencies of broadness in the non-HLA antibody profile. The x-axis displays the number of antibodies for the period 1–2 years post-transplant. The y-axis shows the relative frequency, expressed as the percentage of patients exhibiting the respective number of positive antibodies, bars represent categories constructed in 5-unit increments. **(B)** Distribution of the relative frequencies of strength in the non-HLA antibody profile. The x-axis displays the strength, defined as the sum of all non-HLA antibody ratios >1 per patient for the period 1–2 years post-transplant. The y-axis shows the relative frequency, expressed as the percentage of patients exhibiting the respective strength value, bars represent categories constructed in 20-unit increments.

Within the late ABMR group, one subgroup showed broad and intense antibody responses, while another had only few positive antibodies. In contrast, antibody diversity in controls appeared more evenly distributed, though individual outliers with high antibody intensity were noted in both groups, (Figure 2). Pre-transplant levels of individual detectable non-HLA antibodies, stratified by those who underwent dialysis prior to KTx and those receiving a preemptive transplant (Supplementary Figure S1), revealed no significant impact of dialysis on average broadness of non-HLA antibodies (p = 0.8).

### Association Between Non-HLA Antibodies and Late Antibody Mediated Rejection

To assess whether variables of non-HLA immunity may serve as potential predictors for the occurrence of late ABMR, we performed multiple logistic regression analyses. Univariable analyses revealed no statistically significant differences in baseline clinical characteristics between the late ABMR and control groups, including sex, age at transplantation, dialysis prior to transplantation, or retransplantation. Donor type (living vs deceased) approached significance (p = 0.07) but was excluded from multivariable models due to the absence of living donors in the late ABMR group. Cumulative non-HLA antibody measures (broadness and strength) showed no significant association with late ABMR either pre- or 1–2 years post-transplant, (Table 3). Multiple logistic regression based on the same variables confirmed these findings. Two models were conducted: one with pre-transplant cumulative variables and baseline characteristics, and another using the same variables 1–2 years post-transplant, (Table 4). After model reduction by retaining only variables from the initial models with p-values ≤0.17, only cumulative broadness at 1–2 years post-transplant approached significance (p = 0.09), showing a negative association, (Supplementary Table S3). Due to the absence of HLA-DSA positive individuals in the control group at the 1–2 years post-transplant interval, associations between DSA status and late ABMR could not be assessed using regression analysis.

**TABLE 3:** Tests of association between the event late ABMR and baseline characteristic variables as well as cumulative broadness and cumulative strength measured pre-transplant and 1–2 years post-transplant respectively.

Test of association	Variable	p-value
Chi-squared test of independence	Sex	0.559
Chi-squared test of independence	Donor type	0.067
Welch two sample t-test	Age at KTX	0.577
Chi-squared test of independence	Re-KTX	0.480
Chi-squared test of independence	Dialysis prior to KTX	1
Welch two sample t-test	Cum. broadness pre-transplant	0.225
Welch two sample t-test	Cum. strength pre-transplant	0.331
Welch two sample t-test	Cum. broadness 1–2 years post-transplant	0.427
Welch two sample t-test	Cum. strength 1–2 years post-transplant	0.932

**TABLE 4:** Multivariate logistic regression analyses for estimating the relationships between different patient factors and the event late ABMR, model a) uses pre-transplant, model b) 1–2-year post-transplant antibody broadness and strength.

Model a)	Reference	Estimate	SE	Statistic	p-value
Intercept		−1.125	0.86	−1.30	0.19
Sex (female)	(Male)	0.982	0.68	1.45	0.15
Age at KTX		0.001	0.06	0.02	0.98
Re-KTX (yes)	(No)	0.836	0.75	1.12	0.26
Dialysis prior to KTX (yes)	(No)	−0.192	0.61	−0.31	0.75
Cumulative broadness		−0.027	0.04	−0.74	0.46
Cumulative strength		−0.001	0.01	−0.06	0.95

Model b)	Reference	Estimate	SE	Statistic	p-value
Intercept		−0.748	0.93	−0.80	0.42
Sex (Female)	(Male)	0.684	0.66	1.04	0.30
Age at NTX		−0.003	0.06	−0.06	0.95
ReKTX (Yes)	(No)	0.595	0.69	0.86	0.39
Dialysis before NTX (Yes)	(No)	−0.417	0.60	−0.69	0.49
Broadness		−0.058	0.04	−1.65	0.10
Total strength		0.011	0.01	1.41	0.16

Individual non-HLA variables were analyzed to evaluate their potential association with late ABMR. To address model overfitting and estimation instability due to the high number of predictors (60 non-HLA antibodies) and limited sample size (n = 75), a multiple logistic regression with Lasso regularization was applied. Cross-validation was used to determine the regularization parameter. This analysis was performed separately for non-HLA antibodies assessed at the pre-transplant and 1–2 years post-transplant timepoints. For pre-transplant non-HLA profiles, Lasso regression excluded 51 of 60 predictors. Backward selection using the Akaike Information Criterion (AIC) identified a reduced model including three non-HLA antibodies with potential relevance for late ABMR: SNRPB2 (positive coefficient, p = 0.03), ARGN (negative, p = 0.08), and ARHGDIB (negative, p = 0.09), the latter two showing significance only at the 10 percent level. For post-transplant profiles, 52 of 60 predictors were excluded by Lasso regression. AIC-based backward selection retained five non-HLA antibodies. Of those, CGB5 (negative, p = 0.02), ACTIN (positive, p = 0.05), and COLLAGEN V (negative, p = 0.08) showed potential relevance (Table 5). Complete Lasso results for both timepoints are provided in Supplementary Table S4.

**TABLE 5:** Multiple logistic regression model with Lasso regularization after backward model selection using the Akaike Information Criterion (AIC) was performed.a) uses pre-transplant, model b) 1–2-year post-transplant antibody broadness and strength.

Model a)	Estimate	SE	Statistik	p-value
Intercept	−1.001	0.432	−2.318	0.020
AGRN	−1.438	0.833	−1.726	0.084
ARHGDIB	−0.828	0.482	−1.717	0.086
CXCL9	0.121	0.088	1.373	0.170
SNRPB2	0.980	0.452	2.167	0.030

Model b)	Estimate	SE	Statistik	p-value
Intercept	−0.167	0.521	−0.320	0.749
ACTIN	0.979	0.501	1.955	0.051
ARHGDIB	−0.343	0.257	−1.332	0.183
CGB5	−2.589	1.117	−2.318	0.020
COLLAGEN V	−1.964	1.114	−1.762	0.078
IFNG	0.427	0.449	0.950	0.342

### Development of Non-HLA Antibodies Over Time

**FIGURE 3:**
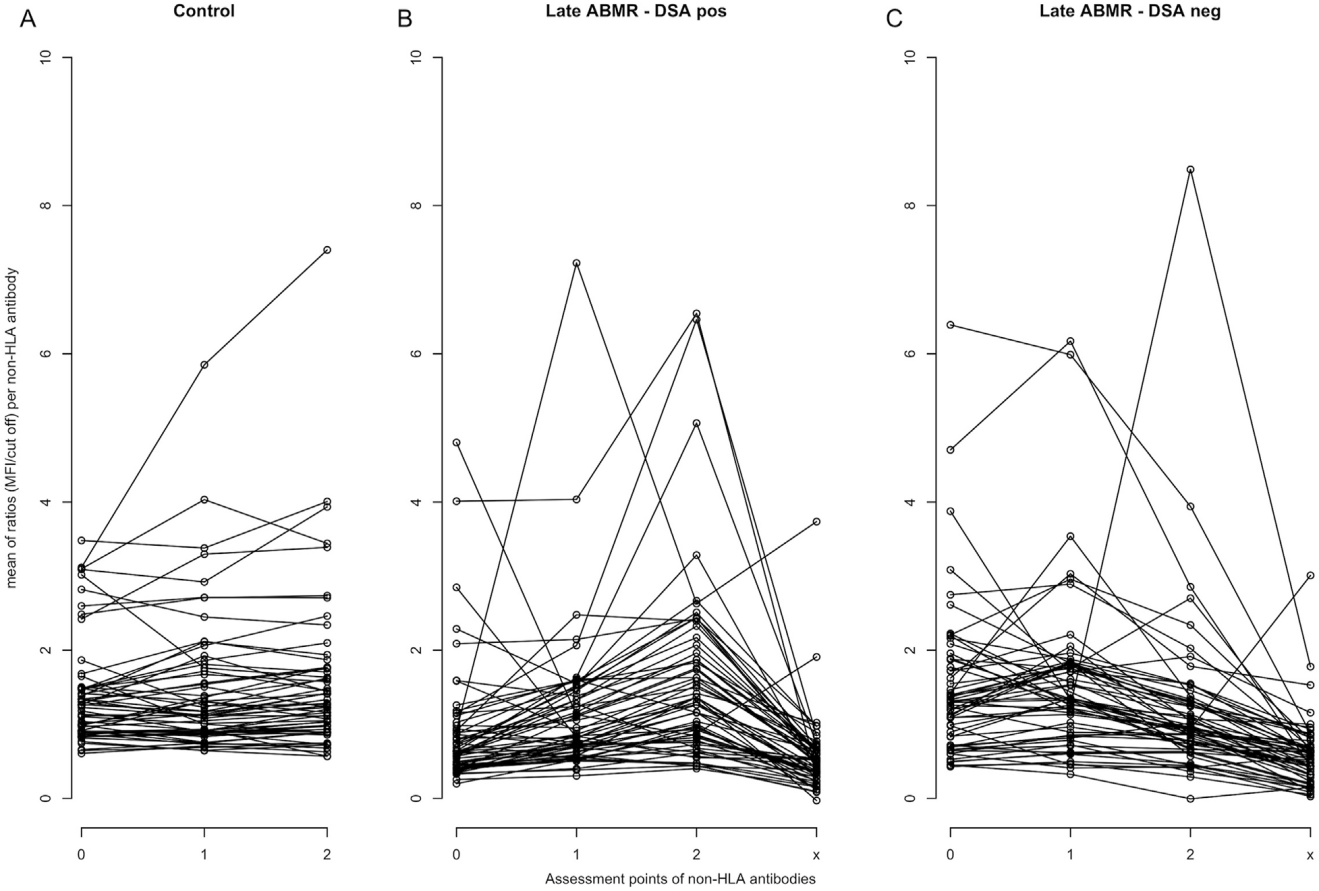
Trajectory of individual non-HLA antibody profiles over time. Each line represents the mean MFI-to-cut-off ratio over all patients for a specific non-HLA antibody. Antibody trajectories are shown separately for the control cohort **(A)**, late ABMR DSA-positive **(B)**, and late ABMR DSA-negative **(C)**. Assessment points on the x-axis represent: 0 = pre-transplantation, 1 = 1–12 months post-transplantation, 2 = 1–2 years post-transplantation, and x = time of rejection diagnosis. The y-axis indicates the mean MFI-to-cut-off ratio.

Pearson correlations of the same non-HLA antibodies pre- and 1–2-year post-transplant revealed strong correlations (r >0.8, p < 0.05) for 3 antibodies in the ABMR DSA-positive group, 9 in the DSA-negative group, but none in controls. Moderate correlations (r = 0.4–0.8, p < 0.05) were found for 11 antibodies in the ABMR DSA-positive, 8 in the DSA-negative, and 26 in the control group (Supplementary Table S5). At 1–2 years post-transplant, correlations between pairs of different non-HLA antibodies revealed strong correlations (r >0.8, p < 0.05) in 825 pairs (46.6%) in the late ABMR DSA-positive group, 720 pairs (40.7%) in the DSA-negative group, compared to 33 pairs (1.9%) in controls, Supplementary Figures S2-S4. Dataset analysis showed that none of the non-HLA antibodies included in the panel were completely absent in all pre-transplant samples. Analysis of the trajectory of the non-HLA antibody profile indicated distinct antibody dynamics between ABMR and controls. In the late ABMR cohort, particularly those with concomitant HLA-DSA, non-HLA antibody levels increased post-transplant and declined in at the onset of rejection. The control cohort showed a more stable profile, characterized by less fluctuation, (Figure 3), apart from Interleukin-21 (IL-21), increasing gradually and reaching the highest mean ratio in the control group (mean: 7.44), compared to lower levels in the ABMR DSA-positive (2.44) and DSA-negative (2.34) groups, (p < 0.05). This difference was primarily driven by five outliers in the control group, (Supplementary Figures S5, S6). IL-21 was not among the most prominent antibodies in late ABMR cohorts. Given the low prevalence of DSA in the control group pretransplant (2/54) and post-transplant (0/54), further stratification by DSA status was not pursued in the control cohort.

**FIGURE 4:**
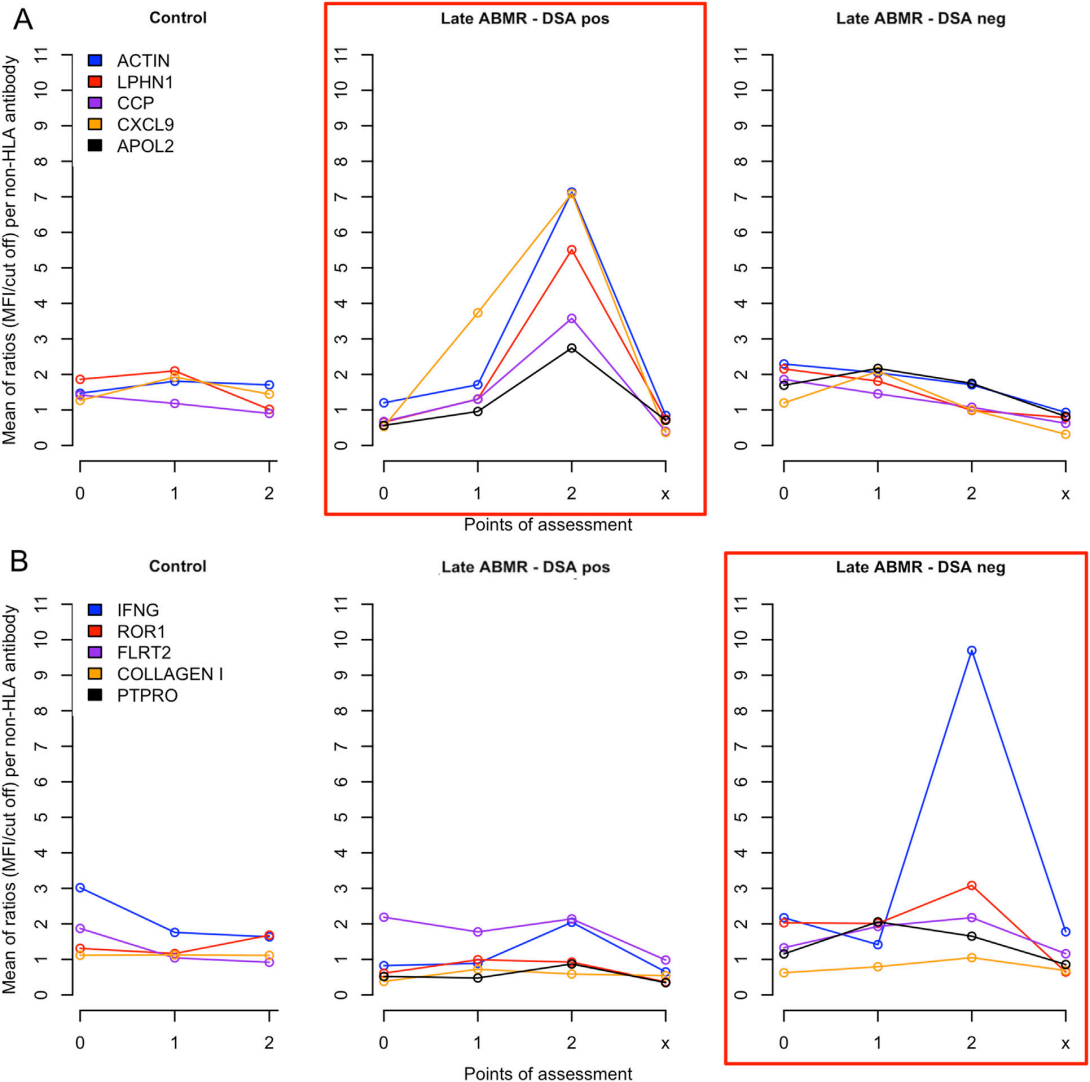
Temporal evolution of the five non-HLA antibodies showing the strongest relative increase in patients with late ABMR. **(A)** Five non-HLA antibodies showing the strongest relative increase in patients with late ABMR -DSA-positive at the time of rejection. The central panel (highlighted in red) shows the course of the levels of antibodies against ACTIN, LPHN1, CCP, CXCL9, and APOL2 in the group late ABMR DSA-positive (Late ABMR–DSA pos). For comparison, the same antibodies are displayed in the control (left panel) and DSA-negative ABMR group (right panel, Late ABMR-DSA neg). **(B)** Five non-HLA antibodies showing the strongest relative increase in patients with late ABMR–DSA-negative at the time of rejection. The right panel (highlighted in red) shows the course of the levels of antibodies against IFNG, ROR1, FLTR2, COLLAGEN I, PTPRO in the group late ABMR DSA-negative (Late ABMR–DSA neg). For comparison, the same antibodies are displayed in the control (left panel) and DSA-positive ABMR group (central panel, Late ABMR–DSA pos). For both assessments the x-axis represents antibody measurement time points: 0 = pre-transplantation, 1 = 1–12 months post-transplantation, 2 = 1–2 years post-transplantation, and x = time of rejection diagnosis (not for the control cohort). The y-axis indicates the mean MFI-to-cut-off ratio.

The five non-HLA antibodies with the strongest increase over time were identified separately for each study group, (Figure 4). Between pre-transplant phase and 1–2 years post-transplant, IFNG and ROR1 showed the highest mean increase between in the ABMR DSA-negative group. CXCL9 showed a similar temporal pattern in the ABMR DSA-positive group with significant differences to the other cohorts. However, after exclusion of one outlier with markedly elevated CXCL9 levels, the difference between groups was no longer statistically significant. From the pre-transplant phase to the time of rejection CXCL11 showed the most distinct upward trend in the DSA-positive ABMR group, without showing significance.

## Discussion

To our knowledge, this is the first explorative study assessing a comprehensive range of non-HLA antibodies in pediatric KTx recipients encompassing both pre- and post-transplantation periods. We demonstrated that 53% of pediatric patients with CKD stages 5–5D, listed for KTx, showed non-HLA autoantibodies prior to transplantation. This indicates a substantial degree of non-HLA autoantibody immunity even prior to KTx, which did not significantly change over time. Despite this, no statistically significant association between the presence of non-HLA antibodies and the development of ABMR was observed during a median follow-up of 4.83 years (IQR = 3.08–6.96).

Vascular damage, particularly in the kidneys themselves, caused by chronic kidney injury and its systemic consequences, appears to be a major factor in the pre-transplant development of non-HLA antibodies, functioning as alloantibodies. Notably, dialysis prior to transplantation did not exert a significant impact on the burden of non-HLA antibodies. This may be at least partly explained by the finding that advanced cardiovascular damage, such as vascular calcifications, is predominantly observed in adolescents and young adults within the pediatric population undergoing dialysis [41]. The observation that none of the tested non-HLA antibodies were consistently absent in all pre-transplant samples suggests that they do not represent purely alloimmune responses. Contrary to studies in adult KTx patients [42], our pediatric cohort did not show a decrease in non-HLA antibody levels post-transplant despite immunosuppressive therapy. Moreover, the highest proportion of at least moderate correlations between pre- and post-transplant non-HLA antibody levels was observed in controls (43%), suggesting a sustained antibody profile independent of ABMR development.

Several studies have indicated the potential involvement of non-HLA antibodies in the pathogenesis of ABMR in adult KTx patients [16, 17, 43]. However, there are currently no established diagnostic protocols for routine testing of non-HLA antibodies in KTx patients, thus precluding a conclusive assessment of their individual clinical relevance and their overall burden when screening for ABMR.

In this present study, the aggregated non-HLA antibody burden, defined by broadness and strength, showed no predictive value for the development of late ABMR. A negative association between post-transplant antibody broadness and ABMR risk was observed, yet the result narrowly failed to reach statistical significance (p = 0.08), highlighting limitations of relying on cumulative measures in capturing immune dynamics associated with ABMR. This prompted a focused analysis of individual non-HLA antibodies, assessed both pre- and post-transplant, considering the already elevated pre-transplant antibody burden.

Pre-transplant antibodies against SNRPB2 were significantly positively associated with late ABMR, prior only associated with the recurrence of focal segmental glomerulosclerosis in kidney transplants [44]. Conversely, antibodies against ARGN and ARHGDIB showed negative marginal associations with ABMR occurence, (p < 0.1), although prior studies linked these antibodies to graft dysfunction and transplant glomerulopathy, warranting cautious interpretation [45, 46]. Post-transplant, antibodies against ACTIN were significantly positively associated with late ABMR. While a direct link between anti-ACTIN antibodies and graft loss has not yet been established, related cytoskeletal mechanisms have been implicated in chronic ABMR pathogenesis. Notably, increased phosphorylation of actin-associated proteins such as ARPC2 has been observed in peripheral blood mononuclear cells from patients with chronic ABMR, suggesting possible cytoskeletal dysregulation in immune cells [47, 48]. Although anti-CGB5 antibodies have previously been associated with post-transplant recurrence of FSGS(44), our findings indicate a negative association with late ABMR, leaving their broader role in transplant immunology to be further clarified.

Due to the high pre-transplant non-HLA antibody burden and to overcome the limitations of analyzing single time-points, a trajectory-based analysis was applied to capture non-HLA antibody dynamics. They revealed distinct temporal patterns between patients with and without late ABMR. In ABMR cohorts, particularly those with HLA-DSA, an initial post-transplant increase in parts of the non-HLA repertoire suggested subclinical alloimmune priming. This was followed by a marked decline at the time of rejection, coinciding with manifest histopathological injury. The observed drop in circulating non-HLA antibodies may reflect compartmentalization of immune mediators into the graft, potentially accumulating at the site of rejection. Concurrent assessment of these mediators and their antibodies in graft tissue or urine could offer further insight into their spatial dynamics and potential pathogenic roles. Conversely, controls exhibited more stable antibody levels, except for a few distinct outliers, seen in a marked post-transplant increase in anti-IL-21 antibodies, predominantly driven by five strong outliers. IL-21 has been implicated in pro-inflammatory pathways in transplantation [49–51]. The observed anti–IL-21 pattern with extreme levels in single patients could reflect an adaptive response to elevated IL-21 levels—potentially indicating subclinical alloimmune activity—or a modulatory mechanism interfering with IL-21–mediated signaling.

The five most prominently increasing non-HLA antibodies varied between ABMR subgroups, suggesting that HLA-DSA status may influence distinct trajectories of antibody development and reflect divergent underlying immune activation pathways. In DSA-negative ABMR, anti-IFNG and anti-ROR1 antibodies exhibited the strongest increases, whereas anti-ACTIN and anti-CXCL9 antibodies rose most prominently in the DSA-positive group. Despite the within-group increases, intergroup differences were not statistically significant, partly due to individual outliers. Anti-CXCL9 showed a transiently significant association, primarily driven by a single DSA-positive case with markedly elevated levels, thus precluding definitive conclusions regarding its predictive relevance, underscoring the need for validation in larger, balanced cohorts.

An objective of this study was to determine whether broad-spectrum screening could identify clinically relevant non-HLA antibodies in pediatric kidney transplant recipients, independent of confounding factors. The predominance of CAKUT limited the detection of disease-specific antibody patterns. Moreover, post-transplant biopsies in controls revealed diverse histopathologies. Modifications in immunosuppression due to CNI toxicity or viral infections may have further contributed to interindividual variability. While our data suggest that HLA-DSA status may influence the evolution of non-HLA antibody responses as reflected in varying correlation patterns, no consistent differences in the most increasing non-HLA antibodies between DSA-positive and DSA-negative patients were observed.

A strength of our study is the analysis of both pre- and post-transplant non-HLA antibody immunity. This provided new insights into the impact of alloimmunity and autoimmunity on the development of the tested non-HLA antibody profile in pediatric patients. The high pre-transplant antibody burden observed underscores the relevance of autoimmune mechanisms in the development of non-HLA antibodies.

Our study is limited by several factors, most notably the retrospective study design, the relatively small number of patients exhibiting ABMR and the sensitivity of the analysis to individual outliers. The latter was partially addressed through targeted outlier analyses and potential impacts on results were reported. The etiology of ABMR is multifactorial, which presents a challenge in identifying individual risk parameters for developing an ABMR. Although patient age was not identified as an independent risk factor in the present group, future studies should also account for factors such as adherence to immunosuppressive therapy, particularly in adolescents.

Large-scale studies are necessary to determine the prognostic relevance of specific non-HLA antibodies in pediatric renal transplant patients. Given the high prevalence of non-HLA antibodies already prior to transplantation, more studies on non-HLA alloimmunity in renal-healthy children and those with renal insufficiency should be conducted. This would forward more detailed information regarding specific alterations in the antibody profile under investigation.

## Data Availability

The original contributions presented in the study are included in the article/supplementary material, further inquiries can be directed to the corresponding author.
