# Supplementary figures and images for: Development of Non-HLA Antibodies and Their Association With Antibody-Mediated Rejection in Pediatric Kidney Transplant Recipients
Source: Transpl Int. 2025 Aug 7;38:14463. doi: 10.3389/ti.2025.14463 (PMC12369655; doi:10.3389/ti.2025.14463)

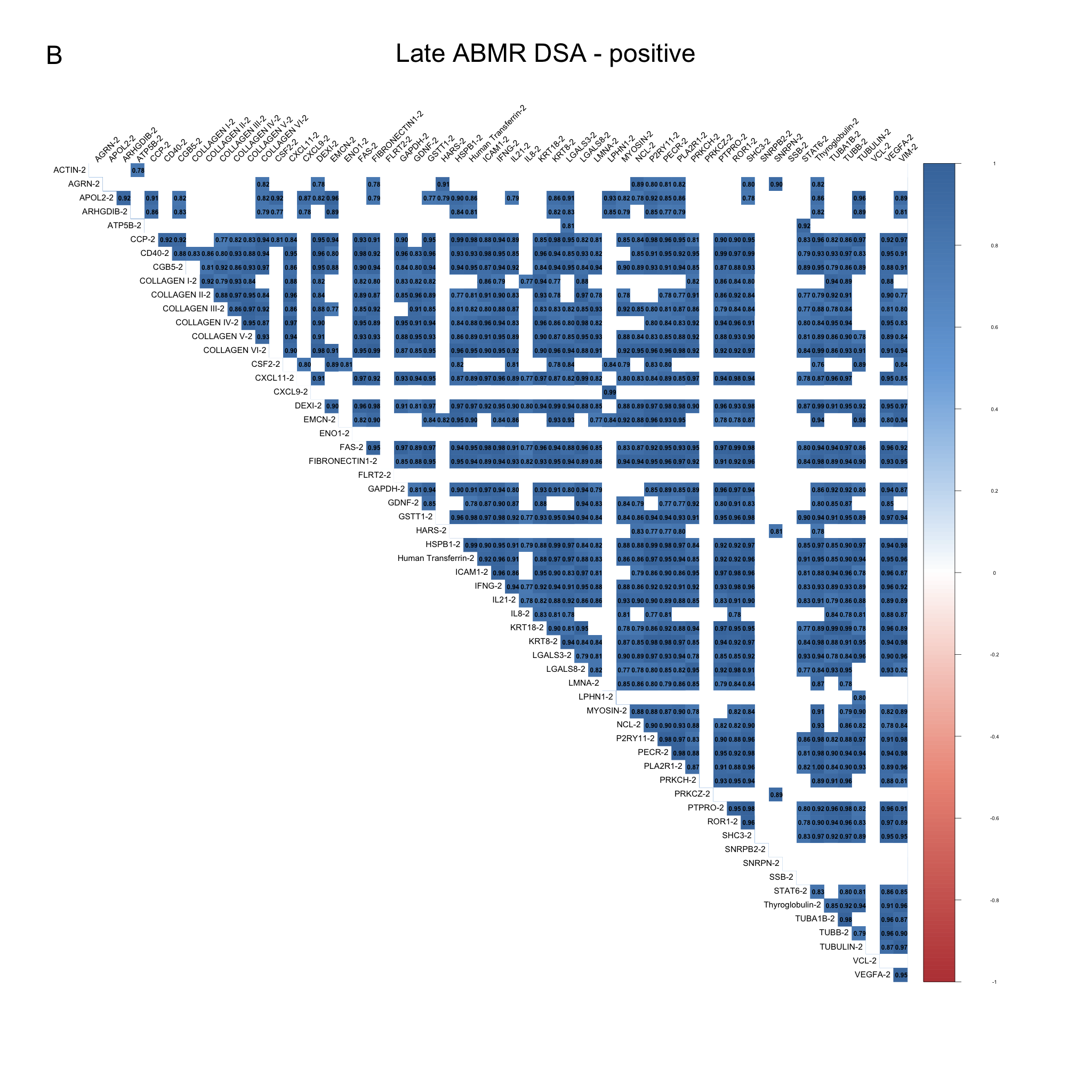

Supplement: Supplementary file 1 [file Image3.tiff]

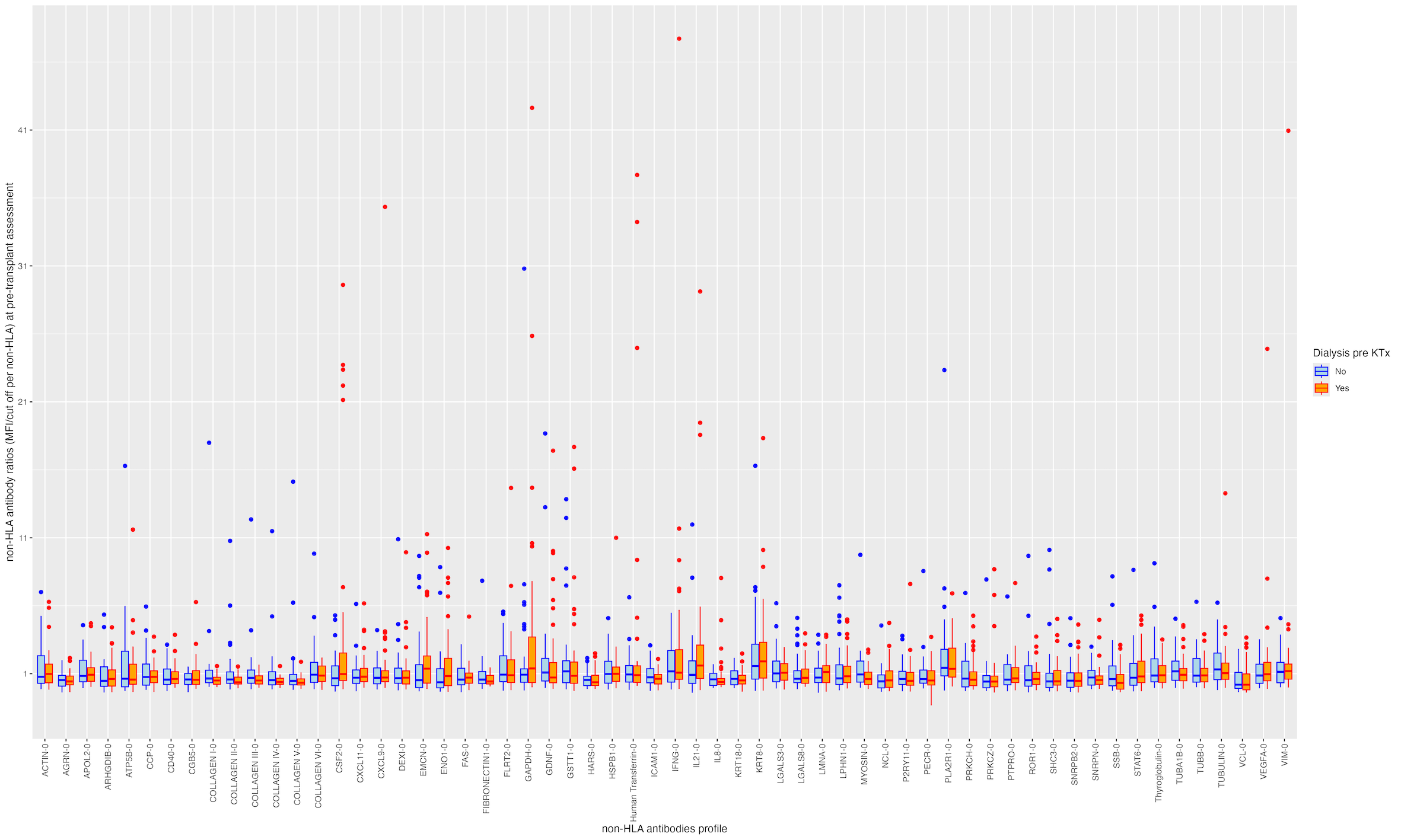

Supplement: Supplementary file 2 [file Image1.tiff]

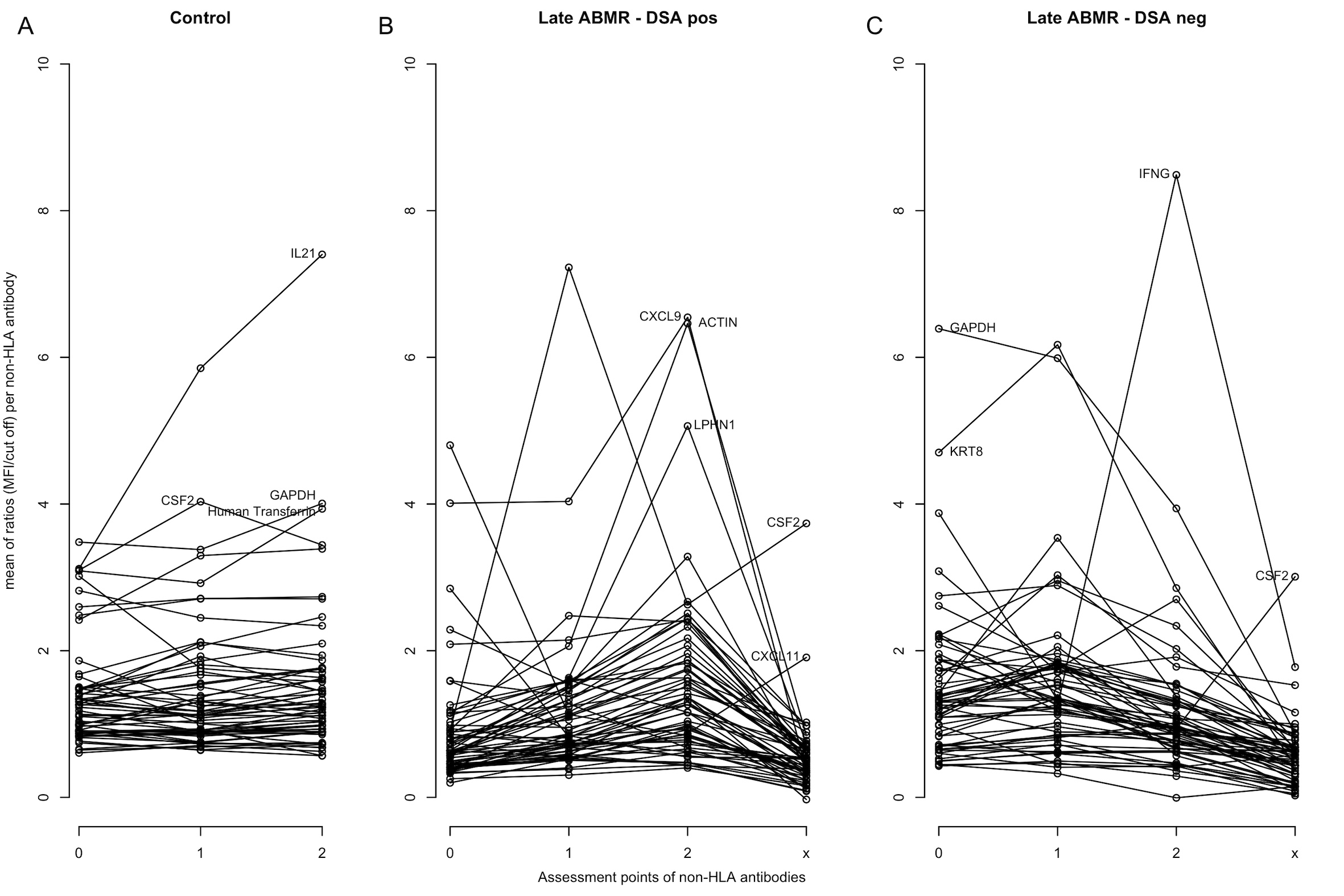

Supplement: Supplementary file 3 [file Image5.jpeg]

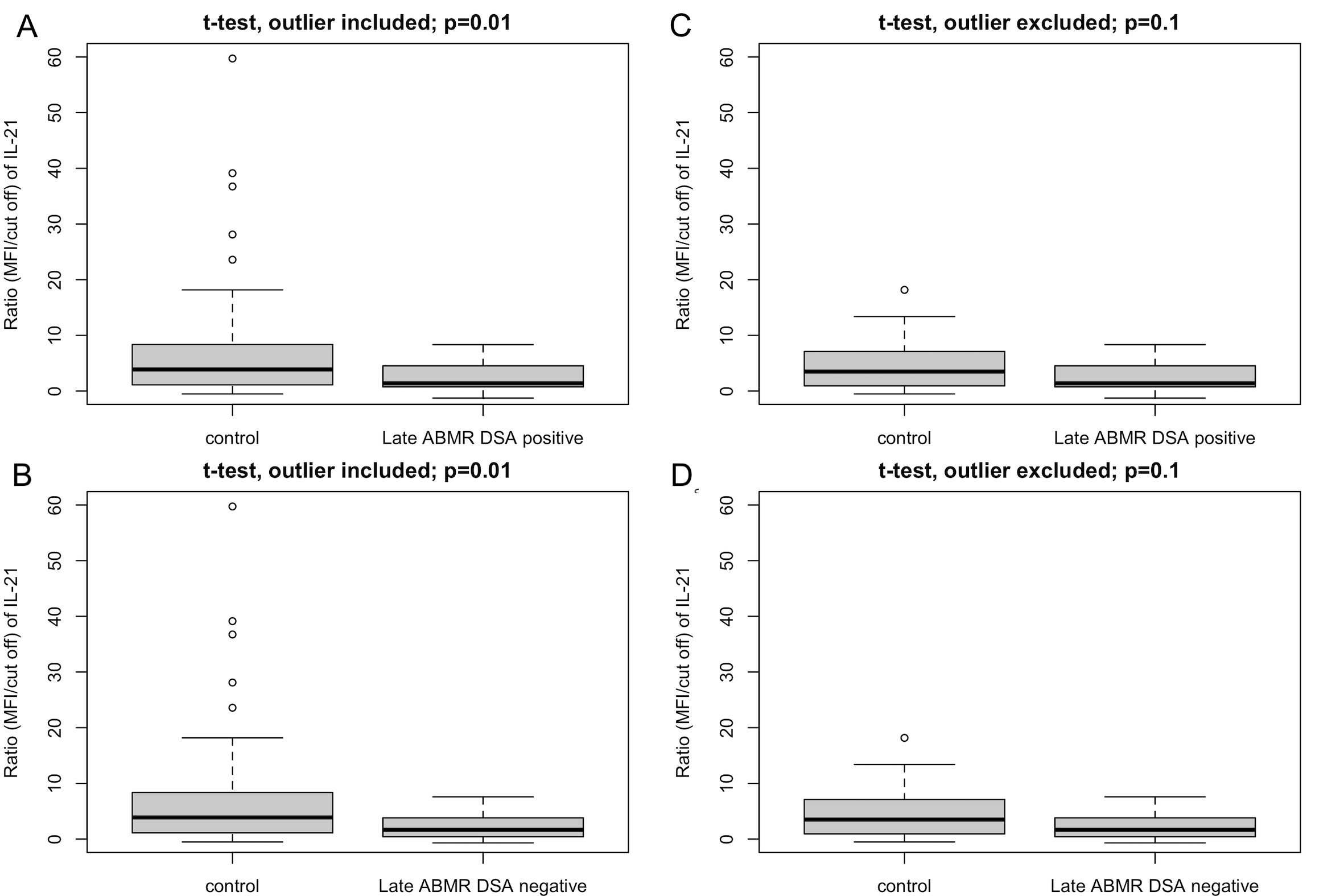

Supplement: Supplementary file 4 [file Image6.tiff]

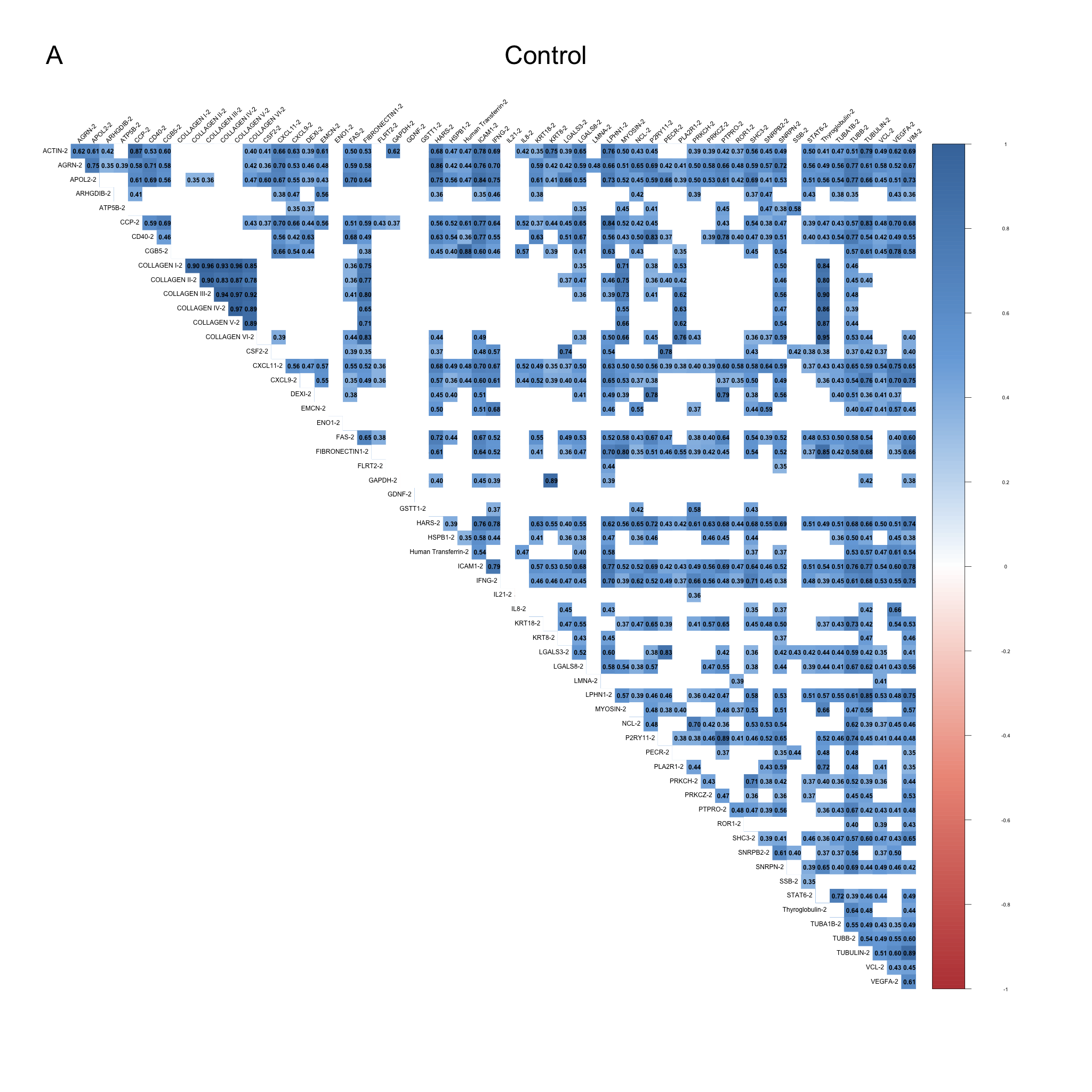

Supplement: Supplementary file 5 [file Image2.tiff]

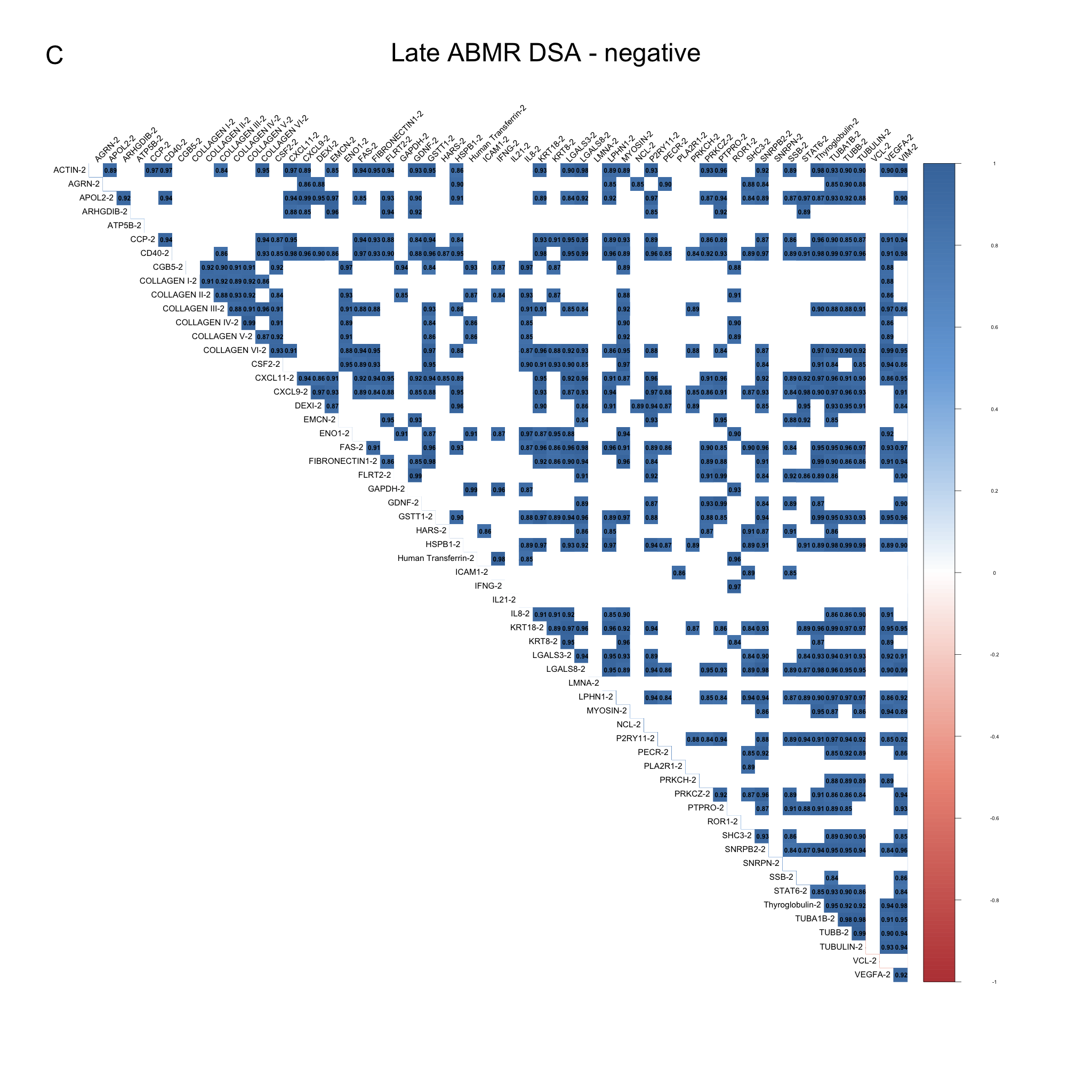

Supplement: Supplementary file 6 [file Image4.tiff]
