# Supplementary material for: Development of Non-HLA Antibodies and Their Association With Antibody-Mediated Rejection in Pediatric Kidney Transplant Recipients
Source: Transpl Int. 2025 Aug 7;38:14463. doi: 10.3389/ti.2025.14463 (PMC12369655; doi:10.3389/ti.2025.14463)
Supplement: Supplementary file 7 [file DataSheet1.docx]

**Supplementary Material:** **Development of non-HLA antibodies and their association with antibody-mediated rejection in pediatric kidney transplant recipients**

**Figure S1**

**Pre-transplant levels of detectable non-HLA antibodies, stratified state of dialysis prior to KTx.**

The Boxplot depicts the ratios of single non-HLA antibodies at period "-0," which refers to the pretransplant status of the patient cohort. The cohort was divided into two groups: those who received dialysis prior to KTx (orange columns) and those who did not receive dialysis prior to KTx (blue columns).

## Figure S2 A-C

**Pearson correlation analysis between different non-HLA antibodies 1–2 years post-transplant.**

Pairwise Pearson correlations were calculated between the ratios (MFI/cut-off) of 60 non-HLA antibodies (n = 1770 unique pairs) for serum samples obtained 1–2 years post-transplant. Correlation matrices are shown separately for the control cohort (A), the late ABMR DSA-positive cohort (B), and the late ABMR DSA-negative cohort (C). Color intensity indicates the strength of positive correlation coefficients; only statistically significant correlations (p < 0.05) are displayed. The number of significant correlations was 1081 in the control cohort, 1154 in the late ABMR DSA-positive cohort, and 720 in the late ABMR DSA-negative cohort. Negative correlations did not reach statistical significance and are therefore not shown. Strong positive correlations (r > 0.8) were observed in 33 pairs (1.9% of all correlations) in the control cohort, 825 pairs (46.6%) in the late ABMR DSA-positive cohort, and 720 pairs (40.7%) in the late ABMR DSA-negative cohort. Data are based on all available serum samples collected at the 1–2-year post-transplant assessment.

**Figure S3**

**Trajectory of individual non-HLA antibody profiles over time - with line labels.**

Figure S2 displays the same data as shown in Figure 3, but includes name labels on the highest-ranking lines. Each line represents the mean MFI-to-cut-off ratio over all patients for a specific non-HLA antibody. Antibody trajectories are shown separately for the control cohort (panel A), late ABMR with DSA (panel B), and late ABMR without DSA (panel C). Assessment points on the x-axis represent: 0 = pre-transplantation, 1 = 1–12 months post-transplantation, 2 = 1–2 years post-transplantation, and x = time of rejection diagnosis. The y-axis indicates the mean MFI-to-cut-off ratio.)

**Figure S4**

**Comparison of IL-2 antibody levels between control and late ABMR groups, stratified by DSA status (DSA-positive and DSA-negative), using an unpaired two-tailed Student’s t-test.** The left panels (A,B) include five outliers (depicted as individual points) A) comparison between control and ABMR DSA-positive, B) comparison between control and ABMR DSA-negative. The right panels (C,D) show the results after exclusion of these outliers C) comparison between control and ABMR DSA-positive, D) comparison between control and ABMR DSA-negative. Data are presented as mean ± standard deviation. A p-value <0.05 was considered statistically significant.

**Table S1**

Predominant histological diagnoses from indication biopsies in the control group (n = 41). In cases with multiple findings, classification was based on the predominant histological lesion.

| **Histological diagnosis** | **Number of**  **patients** |
| --- | --- |
| Tubular injury / atrophy / interstitial fibrosis |  |
| – Mild or not further graded | 11 |
| – Severe | 7 |
| – Diffuse | 2 |
| T cell–mediated rejection | 8 |
| Borderline rejection | 5 |
| Other diagnoses | 6 |
| – Renal pathologies with cystic changes | 2 |
| – Focal segmental glomerulosclerosis (1 with oxalate crystals) | 2 |
| – Thrombotic microangiopathy with endocapillary hypercellularity | 1 |
| – Oxalosis | 1 |
| No pathological findings | 2 |

**Table S2**

Multiple logistic regression including donor type as covariate. model a) uses pre-

transplant, model b) 1–2-year post-transplant antibody broadness and strength.

| **Model a)** | **Reference** | **Estimate** | **SE** | **Statistic** | **p-value** |
| --- | --- | --- | --- | --- | --- |
| Intercept |  | -0.80 | 0.86 | -0.92 | 0.36 |
| Sex (female) | (Male) | 0.81 | 0.71 | 1.14 | 0.25 |
| Age at KTX |  | 0.03 | 0.06 | 0.45 | 0.66 |
| Re-KTX (yes) | (No) | 0.46 | 0.79 | 0.59 | 0.56 |
| donor type (LD) | (DD) | -17.68 | 1749.87 | -0.01 | 0.99 |
| Dialysis prior to KTX (yes) | (No) | -0.31 | 0.63 | -0.49 | 0.62 |
| Cumulative broadness |  | -0.03 | 0.04 | -0.86 | 0.39 |
| Cumulative strength |  | 0.00 | 0.01 | 0.08 | 0.94 |

| **Model b)** | **Reference** | **Estimate** | **SE** | **Statistic** | **p-value** |
| --- | --- | --- | --- | --- | --- |
| Intercept |  | -0.25 | 0.92 | -0.27 | 0.79 |
| Sex (female) | (Male) | 0.76 | 0.72 | 1.06 | 0.29 |
| Age at KTx |  | 0.02 | 0.06 | 0.27 | 0.78 |
| Re-KTX (yes) | (No) | 0.19 | 0.74 | 0.25 | 0.80 |
| donor type (LD) | (DD) | -17.93 | 1729.81 | -0.01 | 0.99 |
| Dialysis prior to KTx (yes) | (No) | -0.54 | 0.63 | -0.86 | 0.39 |
| Cumulative broadness |  | -0.07 | 0.04 | -1.86 | 0.06 |
| Cumulative strength |  | 0.01 | 0.01 | 1.38 | 0.17 |

**Table S3**

Multiple logistic regression after model reduction; by retaining only those variables from the initial regression models (Table 4) with p-values ≤ 0.17.

| **Model a)** | **Reference** | **Estimate** | **SE** | **Statistic** | **p-value** |
| --- | --- | --- | --- | --- | --- |
| Intercept |  | -1.105 | 0.472 | -2.343 | 0.019 |
| Cumulative strength |  | -0.006 | 0.006 | -1.123 | 0.261 |
| Sex (Female) | (Male) | 0.706 | 0.603 | 1.171 | 0.241 |

| **Model b)** | **Reference** | **Estimate** | **SE** | **Statistic** | **p-value** |
| --- | --- | --- | --- | --- | --- |
| Intercept |  | -0.783 | 0.495 | -1.583 | 0.113 |
| Broadness |  | -0.057 | 0.034 | -1.669 | 0.095 |
| Total strength |  | 0.010 | 0.008 | 1.311 | 0.190 |
| Sex (Female) | (Male) | 0.623 | 0.626 | 0.996 | 0.319 |

**Table S4**

The complete Lasso selection results for both timepoints

a) pre-transplant, b) post-transplant

| **Model a)** | **Estimate** | **SE** | **Statistic** | **p-value** |
| --- | --- | --- | --- | --- |
| Intercept | -0.983 | 0.566 | -1.738 | 0.082 |
| AGRN | -0.757 | 0.966 | -0.784 | 0.433 |
| ARHGDIB | -0.496 | 0.507 | -0.978 | 0.328 |
| CGB5 | -0.591 | 0.790 | -0.748 | 0.454 |
| CXCL11 | -0.326 | 0.579 | -0.563 | 0.573 |
| CXCL9 | 0.120 | 0.096 | 1.239 | 0.215 |
| LGALS8 | 0.049 | 0.536 | 0.091 | 0.928 |
| SNRPB2 | 0.905 | 0.456 | 1.986 | 0.047 |
| STAT6 | -0.116 | 0.449 | -0.258 | 0.796 |
| VIM | 0.168 | 0.360 | 0.467 | 0.640 |

| **Model b)** | **Estimate** | **SE** | **Statistic** | **p-value** |
| --- | --- | --- | --- | --- |
| Intercept | 0.178 | 0.652 | 0.273 | 0.785 |
| ACTIN | 0.896 | 0.528 | 1.698 | 0.090 |
| ARHGDIB | -0.303 | 0.269 | -1.125 | 0.261 |
| CGB5 | -2.368 | 1.157 | -2.046 | 0.041 |
| COLLAGEN V | -1.781 | 1.160 | -1.535 | 0.125 |
| IFNG | 0.448 | 0.503 | 0.892 | 0.372 |
| IL21 | -0.111 | 0.118 | -0.943 | 0.346 |
| LMNA | 0.284 | 0.627 | 0.454 | 0.650 |
| PLA2R1 | -0.194 | 0.275 | -0.708 | 0.479 |

**Table S5**

Pearson correlation between non-HLA antibodies in two distinct time periods:

pre-transplant and 1-2 years post KTx, separately analyzed for control, late ABMR DSA-positive and late ABMR DSA-negative.

|  | **Control** |  | **Late ABMR- DSA-positive** | | **Late ABMR DAS-negative** | |
| --- | --- | --- | --- | --- | --- | --- |
| **non-HLA antibody** | **r-coefficient *** | **p-value** | **r-coefficient *** | **p-value** | **r-coefficient *** | **p-value** |
| ACTIN | 0,21 | 0,13 | 0,99 | 0 | 0,74 | 0,04 |
| AGRN | 0,27 | 0,05 | 0,68 | 0,03 | 0,16 | 0,71 |
| APOL2 | 0,15 | 0,29 | 0,38 | 0,28 | 0,55 | 0,16 |
| ARHGDIB | 0,37 | 0,01 | 0,48 | 0,16 | 0,54 | 0,17 |
| ATP5B | 0,6 | 0 | 0,8 | 0,01 | 0,81 | 0,01 |
| CCP | 0,3 | 0,03 | 0,57 | 0,08 | 0,81 | 0,02 |
| CD40 | 0,3 | 0,03 | 0,67 | 0,03 | 0,54 | 0,17 |
| CGB5 | 0,68 | 0 | 0,4 | 0,25 | 0,6 | 0,12 |
| COLLAGEN I | 0,41 | 0 | 0,3 | 0,4 | 0,35 | 0,39 |
| COLLAGEN II | 0,5 | 0 | 0,07 | 0,85 | 0,6 | 0,11 |
| COLLAGEN III | 0,5 | 0 | 0,26 | 0,47 | 0,49 | 0,21 |
| COLLAGEN IV | 0,49 | 0 | 0,17 | 0,63 | 0,63 | 0,09 |
| COLLAGEN V | 0,53 | 0 | 0,11 | 0,76 | 0,42 | 0,3 |
| COLLAGEN VI | 0,39 | 0 | 0,33 | 0,35 | 0,76 | 0,03 |
| CSF2 | 0,35 | 0,01 | -0,31 | 0,38 | -0,36 | 0,38 |
| CXCL11 | 0,51 | 0 | 0,37 | 0,29 | 0,73 | 0,04 |
| CXCL9 | 0,26 | 0,06 | -0,11 | 0,75 | 0,34 | 0,41 |
| DEXI | 0,62 | 0 | 0,28 | 0,43 | 0,19 | 0,65 |
| EMCN | 0,35 | 0,01 | -0,09 | 0,8 | 0,81 | 0,01 |
| ENO1 | 0,65 | 0 | 0,7 | 0,02 | 0,39 | 0,34 |
| FAS | 0,12 | 0,4 | 0,36 | 0,31 | 0,65 | 0,08 |
| FIBRONECTIN1 | 0,2 | 0,16 | -0,03 | 0,94 | 0,69 | 0,06 |
| FLRT2 | 0,28 | 0,04 | 0,73 | 0,02 | 0,53 | 0,18 |
| GAPDH | 0,23 | 0,09 | 0,33 | 0,36 | 0,88 | 0 |
| GDNF | 0,66 | 0 | 0,25 | 0,48 | 0,96 | 0 |
| GSTT1 | 0,48 | 0 | 0,73 | 0,02 | 0,42 | 0,3 |
| HARS | 0,21 | 0,12 | 0,54 | 0,11 | 0,73 | 0,04 |
| HSPB1 | 0,63 | 0 | 0,72 | 0,02 | 0,53 | 0,18 |
| Human Transferrin | 0,57 | 0 | 0,79 | 0,01 | 0,95 | 0 |
| ICAM1 | 0,06 | 0,66 | 0,63 | 0,05 | 0,85 | 0,01 |
| IFNG | 0,11 | 0,44 | 0,27 | 0,46 | 0,09 | 0,84 |
| IL21 | 0,35 | 0,01 | 0,11 | 0,76 | -0,3 | 0,47 |
| IL8 | 0,74 | 0 | -0,26 | 0,46 | 0,41 | 0,32 |
| KRT18 | 0,3 | 0,03 | 0,62 | 0,06 | 0,59 | 0,13 |
| KRT8 | 0,13 | 0,36 | 0,67 | 0,04 | 0,87 | 0,01 |
| LGALS3 | 0,3 | 0,03 | 0,15 | 0,67 | 0,73 | 0,04 |
| LGALS8 | 0,26 | 0,06 | 0,37 | 0,3 | 0,61 | 0,11 |
| LMNA | 0,09 | 0,52 | 0,32 | 0,37 | 0,47 | 0,24 |
| LPHN1 | 0,32 | 0,02 | 0,04 | 0,92 | 0,7 | 0,05 |
| MYOSIN | 0,48 | 0 | 0,19 | 0,6 | 0,72 | 0,05 |
| NCL | 0,52 | 0 | 0,46 | 0,18 | -0,24 | 0,56 |
| P2RY11 | 0,43 | 0 | 0,26 | 0,47 | 0,54 | 0,17 |
| PECR | 0,11 | 0,41 | 0,57 | 0,09 | 0,02 | 0,96 |
| PLA2R1 | 0,4 | 0 | 0,25 | 0,48 | 0,73 | 0,04 |
| PRKCH | 0,53 | 0 | 0,78 | 0,01 | 0,55 | 0,15 |
| PRKCZ | 0,73 | 0 | 0,95 | 0 | 0,25 | 0,55 |
| PTPRO | 0,57 | 0 | -0,06 | 0,86 | 0,38 | 0,35 |
| ROR1 | 0,18 | 0,18 | 0,23 | 0,53 | 0,1 | 0,81 |
| SHC3 | 0,07 | 0,61 | 0,42 | 0,23 | 0,12 | 0,78 |
| SNRPB2 | 0,48 | 0 | 0,92 | 0 | 0,51 | 0,19 |
| SNRPN | 0,31 | 0,02 | 0,78 | 0,01 | 0,4 | 0,33 |
| SSB | 0,54 | 0 | -0,16 | 0,65 | 0,72 | 0,04 |
| STAT6 | 0,31 | 0,02 | 0,16 | 0,67 | 0,12 | 0,78 |
| Thyroglobulin | 0,38 | 0 | 0,09 | 0,79 | 0,52 | 0,19 |
| TUBA1B | 0,18 | 0,2 | -0,15 | 0,68 | 0,54 | 0,17 |
| TUBB | 0,26 | 0,06 | 0 | 1 | 0,54 | 0,17 |
| TUBULIN | 0,27 | 0,05 | -0,18 | 0,61 | 0,62 | 0,1 |
| VCL | 0,57 | 0 | 0,23 | 0,53 | 0,79 | 0,02 |
| VEGFA | 0,44 | 0 | -0,15 | 0,67 | 0,7 | 0,05 |
| VIM | 0,2 | 0,15 | -0,23 | 0,51 | 0,83 | 0,01 |

**Capsule Sentence for: Development of non-HLA antibodies and their association with antibody-mediated rejection in pediatric kidney transplant recipients.**

Assessment of a non-HLA antibody profile in 77 pediatric kidney recipients revealed that high pre-transplant non-HLA antibody burden was not linked to late ABMR, but individual antibodies against SNRPB2, ACTIN, and CGB5 may serve as future risk stratifiers pending validation.
